# Cohabitation with aggressive hosts: description of a new microhisterid species in nests of a ponerine ant with ecological notes

**DOI:** 10.1038/s41598-023-45692-1

**Published:** 2023-10-28

**Authors:** Gabriela Pérez-Lachaud, Nicolas Degallier, Yves Gomy, Manuel Elías-Gutiérrez, Franklin H. Rocha, Jean-Paul Lachaud

**Affiliations:** 1 Departamento de Conservación de la Biodiversidad, El Colegio de la Frontera Sur, Avenida Centenario Km 5.5, Chetumal, Mexico; 2 Paris, France; 3 Nevers, France; 4 Departamento de Ecología y Sistemática Acuática, El Colegio de la Frontera Sur, Avenida Centenario Km 5.5, Chetumal, Mexico; 5 Present Address: Dpto. Apicultura, Campus de Ciencias Biológicas y Agropecuarias, Universidad Autónoma de Yucatán, Mérida, Yucatán México

**Keywords:** Behavioural ecology, Biodiversity, Ecological networks

## Abstract

A new clown beetle species, *Bacanius neoponerae*, is described from Mexican nests of the arboreal ponerine ant *Neoponera villosa* found in the tank bromeliad *Aechmea bracteata*. Adult beetles were found in brood chambers or inner refuse piles, but also outside the ant nests, in decaying organic matter between the bromeliad leaves. No direct interactions between ants and microhisterid beetles could be observed. Several lines of evidence suggest a close relationship either with the ants, specific microhabitats within the ant nests or the bromeliads. Sample site elevation, colony size, monthly rainfall and collecting site were the main variables predicting the association. Almost half of the *N. villosa* colonies were associated with the microhisterids, and larger colonies favored their presence*,* especially during the driest months of the year. Two specimens were found in a nest of another ant species, *Camponotus atriceps,* also inhabiting *A. bracteata*. The new species is the seventh of the genus *Bacanius* reported from Mexico. This is the second time a species of this genus is associated with ants, and the fourth record of a histerid beetle cohabiting with ponerine ants. The small size of these beetles and their very protective body structure may facilitate their cohabitation with such aggressive hosts.

## Introduction

Ant colonies and the resources stored in their nests are targeted by a myriad of organisms that primarily parasitize ants and their brood or their social structure, benefiting from suitable environmental conditions and enemy-free space^1–3^. Symbionts in ant nests establish obligate or facultative relationships with ants and range from highly integrated species, adapted in many ways to live with ants, to poorly integrated species that attempt to escape from ants and rely on fleetness, or species that are very small relative to their host and go unnoticed^3–7^.

Histeridae (Coleoptera) is a very diverse family, both ecologically and morphologically, with over 4700 extant valid species and subspecies distributed worldwide^8,9^. There are ten or eleven subfamilies currently recognized, depending on author^10^. Histerids, commonly known as clown beetles, are primarily generalist predators of immature and adult insects. They are associated with a wide range of habitats and with a variety of substrates including dung, carrion, leaf litter, or other decaying plant and organic matter where their prey are present^11,12^. Multiple histerid lineages have evolved to establish obligate symbioses with various animals, for example, as obligate inhabitants of bird and mammal nests and burrows^13,14^. One species has been found to be a parasite of the cocoons of large Neotropical tarantulas^15^ and many species are frequently found inhabiting social insect nests and hives^13,16,17^. These obligate inquilines often exhibit distinctive defensive morphological modifications as well as behavioral and chemical adaptations that facilitate their symbioses^2,12,14,18^. Clown beetles are characterized by their body shape and structures that protect themselves from attack, such as trichomes and specialized cuticular depressions (fossae) where their appendages can be folded. Obligate myrmecophilous and termitophilous histerids from the subfamily Haeteriinae have long attracted attention^18–21^, but myrmecophilous representatives of other subfamilies have remained understudied. Details of the natural history of most species are scarce. Most described species are known only from the original description and were collected through flight interception, and in some cases with carrion, fruit, or dung baited traps which can provide an indication of their food preferences.

Specifically, there is a lack of information about histerid distribution and ecology in the Neotropics^22^. Here, we describe a new species of *Bacanius* (Dendrophilinae: Bacaniini) and provide information on its ecology and association with ants in southeastern Mexico. Currently, the Bacaniini tribe includes 170 species, most of which are from tropical regions^23^ (N. Degallier, unpubl.). According to Kovarik and Caterino^13^, *Bacanius* is a worldwide distributed genus of microhisterid found in rotten wood, under bark, and in forest litter. Six subgenera of *Bacanius* are recognized and 75 extant species are catalogued^8,9^. Of the 63 genera and 240 species of histerids reported from Mexico^8,24–28^, only six species of *Bacanius* have been reliably cited from this country: *B.* (*Gomyister*) *gomyi* Yélamos from Xalapa, Veracruz^29^; *B.* (*Gomyister*) *subcarinatus* Wenzel & Dybas from Cordoba, Veracruz^22^; *B.* (*Gomyister*) *montanus* Mazur & Sawoniewicz and *B.* (*Gomyister*) *irlanda* Mazur & Sawoniewicz from Lagunas de Montebello and Finca Irlanda, Chiapas^30^; *B.* (*s. str*.) *pusillus* Wenzel (this species is no more considered to be a *Degallierister*: Degallier, unpublished) from Penuela, Tierra Blanca and Tezonapa, Veracruz, and Tamazunchale, San Luis Potosi^31^; and *B.* (*s. str*.) *scalptus* Lewis from Cordoba, Veracruz^22^. Additionally, an unidentified necrophilous species of *Bacanius* was reported from Gómez Farías, Jalisco^32^ but may correspond to one of the species cited above. Biological data concerning *Bacanius* species remain scarce; however, specimens have been collected within superficial soil and decaying rotten logs where, according to Kovarik and Caterino^13^, they probably feed on fungal spores, and at least one ‘necroxenous’ species (species accidentally occurring on carrion) was attracted to carrion-baited traps^32^. To date, association with ants has been reported on a single occasion, involving a single male specimen of an undetermined species of *Bacanius* found in a sample of soil and workers from a refuse deposit of a bivouac of *Eciton dulcium crassinode* Borgmeier in Barro Colorado Island, Panama^33^.

## Material and methods

### Sampling and collection sites

**Figure 1:**
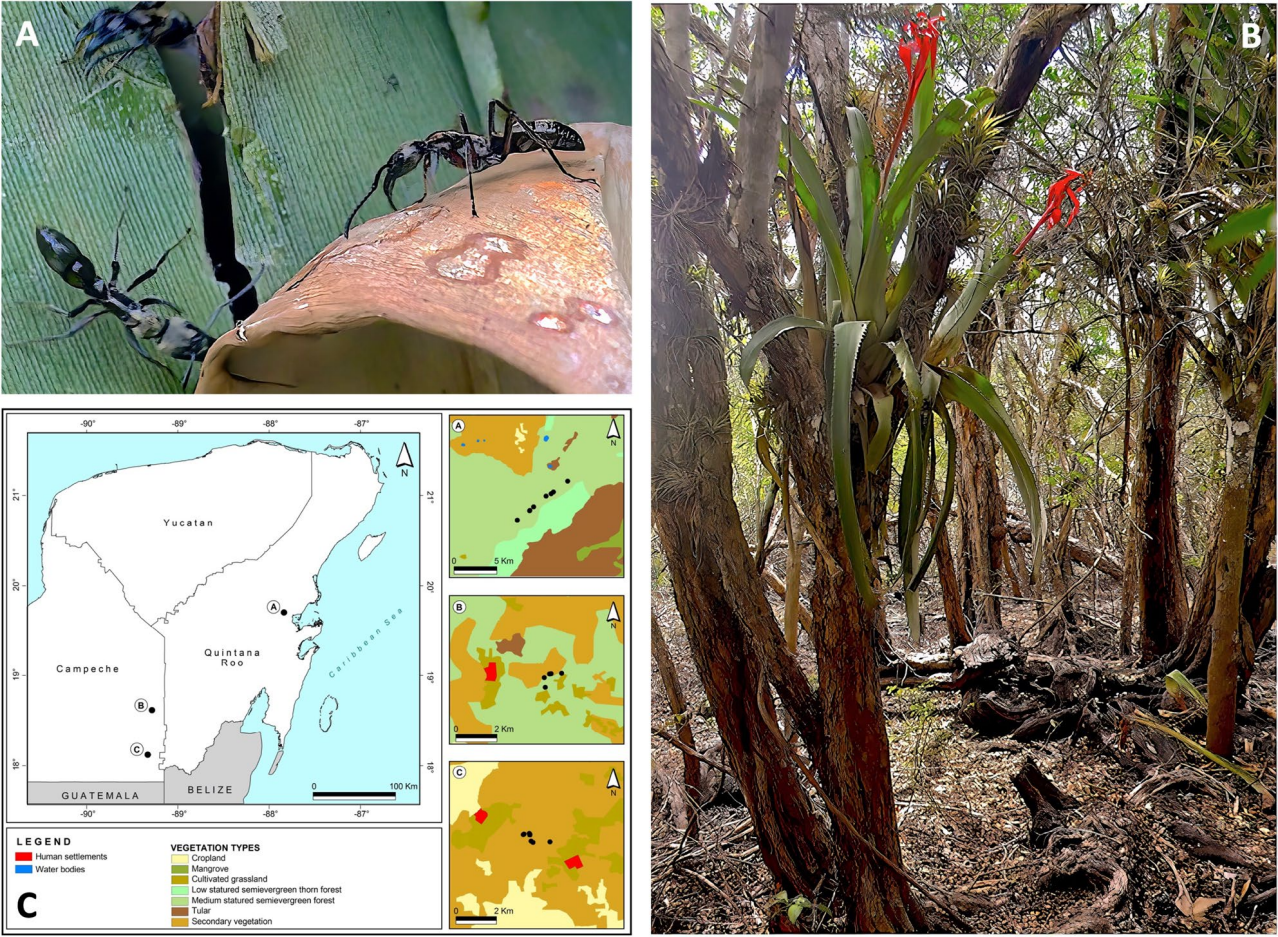
*Neoponera villosa* ants (**A**) with the host plant, *Aechmea bracteata* at Nuevo Becal (**B**), and map of the collection sites (**C**) (Ⓐ Sian Ka’an, Ⓑ Nuevo Becal, Ⓒ Ejido Blasillo). Photos: (**A**) J.-P. Lachaud; (**B**) F.H. Rocha.

The beetles were found in association with the nests of the Neotropical arboreal ant *Neoponera villosa* (Fabricius) (Hymenoptera, Formicidae, Ponerinae) (Fig. 1A) which, in the southern part of the Yucatan Peninsula, Mexico, nests almost exclusively in the epiphytic tank bromeliad *Aechmea bracteata* (Swartz) Grisebach (Poales, Bromeliaceae)^34,35^ (Fig. 1B). Individuals of the new microhisterid species were collected along with the ants as part of a larger project focused on the invertebrate fauna associated with *N. villosa*^35^. A total of 82 *N. villosa* colonies were collected between January 2016 and January 2019, from 3 zones (Nuevo Becal and Ejido Blasillo in the state of Campeche and Sian Ka’an Biosphere Reserve in Quintana Roo), in the southern part of the Yucatan Peninsula (Fig. 1C). The climate of the region is of the “Aw” type according to García^36^, i.e., warm, sub-humid, with rainfall during the summer months and drought in March–April. The arrival of cold fronts from mid-November to February (‘cold’ season) are locally known as “Nortes” (associated with northerly winds) and provide occasional winter precipitation with daily minimum temperatures of 11 °C. Vegetation type of the sampling zones corresponds to tropical forest in different successional stages^37^. Different numbers of colonies were collected per zone and per season due to differences in access facilities and obtaining collection permits. Twenty-five *N. villosa* colonies were collected in the Sian Ka’an Biosphere Reserve (10, 9, and 6 colonies during the dry, the rainy and the cold season, respectively); 24 in Nuevo Becal (10, 4 and 10) and 29 in Ejido Blasillo (12, 6 and 11), accounting for a total of 32 colonies sampled during the dry season, 19 during the rainy season, and 27 during the cold season.

### Ant material identification

According to the keys and descriptions available for the complex of species of the *Neoponera foetida* group^38–40^, the *Neoponera* ants inhabiting most of the epiphytes collected in our study (82 of 84) run to *N*. *villosa*: anterior face of petiole almost straight, vertical, posterior face broadly convex, base of legs reddish, and anterior margin of clypeus concave medially (see Fig. S1 in^41^). Our initial identification was confirmed by J.H.C. Delabie (Laboratorio de Mirmecologia CEPEC/CEPLAC, Itabuna-BA, Brazil) in an earlier work^42^ and further DNA extraction and barcoding^35,43^ (GenBank accession numbers MK779595, MK779597, MK779600, MK779602 and MK779604; see more specifically Fig. S1 in^43^) also showed that the DNA sequences of ants used in our study clustered with all the other publicly available molecular data for *N. villosa*. For the two epiphytes inhabited by *Camponotus*, ant identification was carried out using taxonomic keys or descriptions^44^ and online databases such as AntWeb^45^ and specimens run to *C. atriceps* (Smith) (Formicinae: Camponotini): large size (about 10 mm or more), propodeum narrow and elongate, clypeus with median longitudinal carina, head relatively short and broad, scapes and legs with abundant long, brown or golden, erect setae, mesosoma generally densely hairy.

*Neoponera villosa* is an opportunistic cavity breeder; colonies are established in dead wood, live trees and in the central portion of *A. bracteata* bromeliads where ants build chambers separated by septa made of small debris to house the brood. Colonies were essentially polygynous and contained a mean number of 3 dealate queens, 98 workers, 42 pupae and 41 larvae^35^. Workers measure 12–13 mm^46^ and are very aggressive, with a painful sting. All invertebrates present both in the core of the bromeliads (the ant nests and their immediate border) and those located in the periphery of the nests, including the ants and their brood, were preserved in 96% ethanol, and identified to the lowest possible taxon with available keys. An additional colony of *N. villosa* nesting in a dead branch of a live tree was collected on October 11th, 2022, within the campus of El Colegio de la Frontera Sur, Chetumal, Quintana Roo, and inspected for the presence of any inquiline, particularly for Coleoptera. Two colonies of another ant species (*C. atriceps*), also nesting in *A. bracteata* bromeliads were collected on March 19th, 2018, at Ejido Blasillo and were also inspected for myrmecophiles. Field sampling complied with the current laws of Mexico (collection permit FAUT-0277 from Secretaría del Medio Ambiente y Recursos Naturales—Dirección General de Vida Silvestre granted to GP-L).

### Morphological and molecular studies

Clown beetles were examined using a Leica MZ8 dissecting stereomicroscope (6.3–100×) and a JEOL-JSM6010 scanning electron microscope (SEM). For SEM analysis, specimens were dehydrated in a graded ethanol series from 70 to 100% and let to dry at room temperature or soaked twice in hexamethyldisilazane (© Sigma Aldrich, St Luis, MO, USA) and dry evaporated; they were then fixed onto stubs, and sputter coated with gold before observation. Approximate measurements and ratios of measurements were obtained through a 0.01 mm graduated object micrometer or using SEM images. Photos of the specimens were taken on the imaging station of the Entomology laboratory of the Muséum National d'Histoire Naturelle, Paris (MNHN). Photos were stacked with Zerene Stacker 1.04 (Rik Littlefield, Zerene Systems LLC) and processed in Photoshop®. Methods for specimen dissection, illustration preparation, terminology, body part measurement conventions and abbreviations follow Degallier and Tishechkin^47^.

Five specimens were used for DNA extraction. Due to the small size of the histerids, extraction was performed on the whole specimens using a standard glass fiber method^48^. Polymerase chain reactions were performed to amplify the mitochondrial cytochrome c oxidase subunit I (COI) gene, using the ZplankF1_t1 and ZplankR1_t1 primers originally developed for zooplankton^49^ that work with good success in most groups of invertebrates^50^. PCR protocols follow Montes-Ortiz and Elías-Gutiérrez^51^. The PCR products were visualized on a 2% agarose gel (E-Gel 96 Invitrogen), and positive PCR products were sent for sequencing to Eurofins Genomics, LLC, Kentucky, USA. Sequences were edited using CodonCode v. 3.0.1 (CodonCode Corporation, Dedham, MA, USA) and uploaded to the Barcode of Life Database (BOLD, boldsystems.org, dataset DS-HISTERPY) and to GenBank (www.ncbi.nlm.nih.gov/genbank/; Accession Numbers: OQ706395-98). Specimens were recovered after the lysis step from the glass fiber filter plate and preserved in 96% ethanol.

We added the obtained sequences to a database of published COI barcodes. The resulting matrix included 136 sequences for a total of 22 putative species in five genera belonging to the Dendrophilinae (Table S1). For the ID-tree, the alignment was carried out using MUSCLE^52^ with default settings. This expanded matrix was used to obtain a consensus tree inferred by using the Maximum Likelihood method based on the Tamura-Nei model^53^. The model was selected after the lowest Bayesian Information Criterion (BIC) from the matrix. Initial tree(s) for the heuristic search were obtained automatically by applying Neighbor-Join and BioNJ algorithms to a matrix of pairwise distances estimated using the Maximum Composite Likelihood approach, and then selecting the topology with superior log likelihood value. Support was estimated using 500 bootstrap replications. All analyses were conducted using the Mega V7.0 software.

### Ecology

We further gathered and analyzed data for some environmental and host colony predictor variables that may influence the presence or absence of the histerid in *N. villosa* nests. A classification tree was built with the “repart” library^54^, with bagging as the ensemble method. A total of 78 colonies were considered for statistical analyses (Nuevo Becal: 24 colonies, Ejido Blasillo: 29 colonies, and Sian Ka’an Biosphere Reserve: 25 colonies); four records were not considered as they were from incomplete colonies (only a few workers and no brood). The data set (n = 78 records) considered the following predictors: (a) the sampling zone, altitude, mean monthly temperature, and total monthly precipitation; (b) the colony size and type of colony (queenright or queenless). The Random Forest algorithm (see^55^) establishes the outcome based on the predictions of the decision trees. It predicts by taking the average or mean of the output from various trees, therefore increasing the number of trees increases the precision of the outcome. Significant predictor variables were further explored, and levels were compared with either a parametric test after the Shapiro–Wilk test was used to check if data were normally distributed, or with a non-parametric test. All analyses were performed using R Statistical Software (v4. 1.2^56^).

### Vouchers and nomenclatural act

Extracted specimens of the new species were deposited as vouchers (Catalog Numbers C-2570–2574) along with *N. villosa* workers, at the Arthropoda Collection of El Colegio de la Frontera Sur (ECO-CH-AR) in Chetumal, Quintana Roo, Mexico. The collections where type-material of the new species was deposited are cited in square brackets by the following acronyms:

BMNH: British Museum (Natural History), London, UK.

CHND: Nicolas Degallier collection, Paris, France.

CHYG: Yves Gomy collection, Nevers, France, then Zoologische Staatssammlung München, Munich, Germany.

ECO-CH-AR: Artropoda Collection of El Colegio de la Frontera Sur, Chetumal, Quintana Roo, Mexico.

FMNH: Field Museum of Natural History, Chicago IL, USA.

MNHN: Muséum National d’Histoire Naturelle, Paris, France.

The electronic edition of this article conforms to the requirements of the amended International Code of Zoological Nomenclature, and hence the new name contained herein is available under that Code from the electronic edition of this article. This published work and the nomenclatural act it contains have been registered in ZooBank, the online registration system for the ICZN (https://zoobank.org/urn:lsid:zoobank.org:pub:653C11BD-A9BD-4580-95EF-AB096AA1250E).

## Results

### Description of the new species

Dendrophilinae Reitter, 1909

Bacaniini Kryzhanovskij & Reichardt, 1976

**Figure 2:**
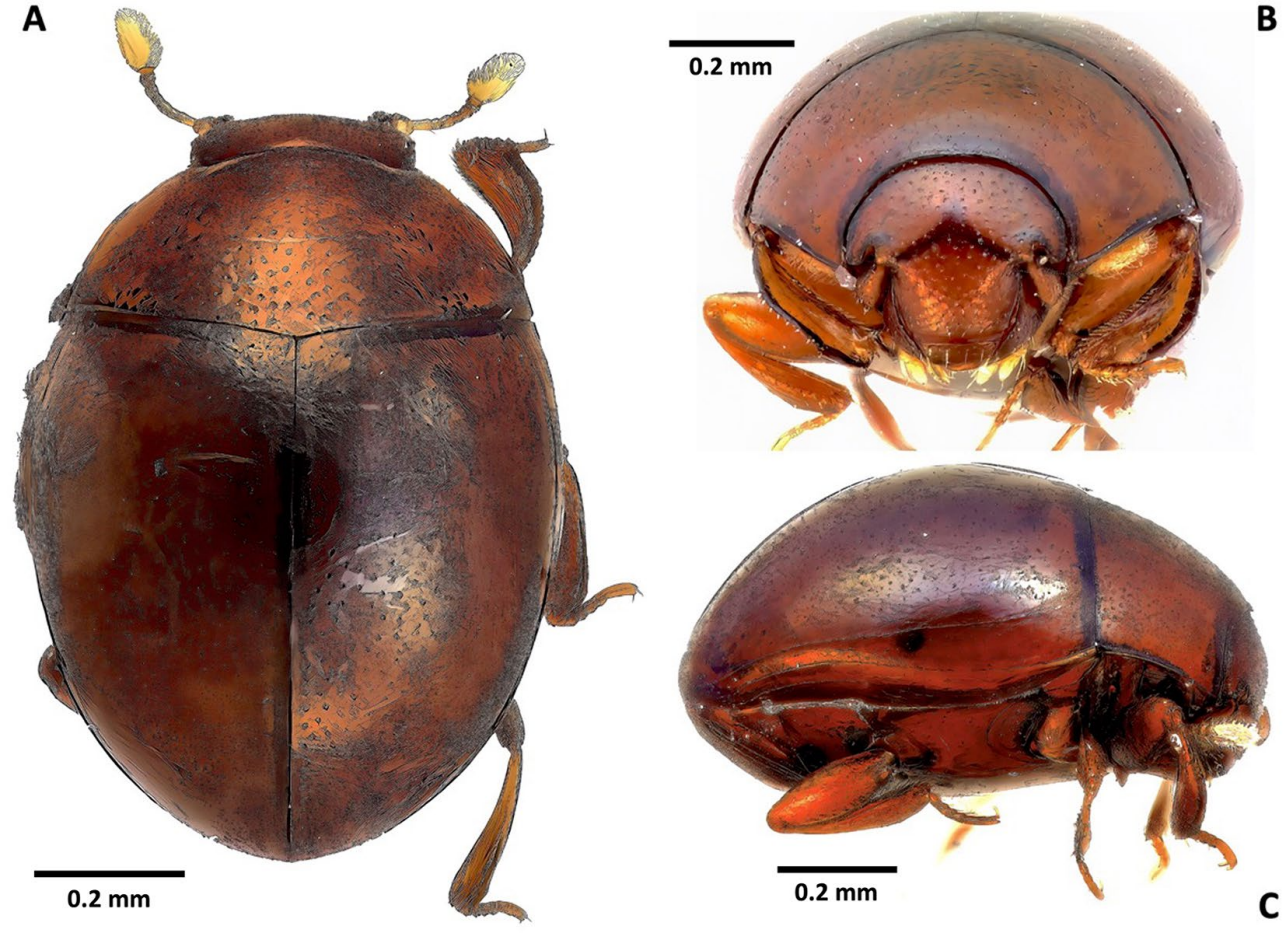
*Bacanius neoponerae* n. sp., habitus dorsal (**A**), frontal (**B**), and lateral (**C**) views. Total length = 1 mm; width = 0.77 mm. Photos: N. Degallier.

**Figure 3:**
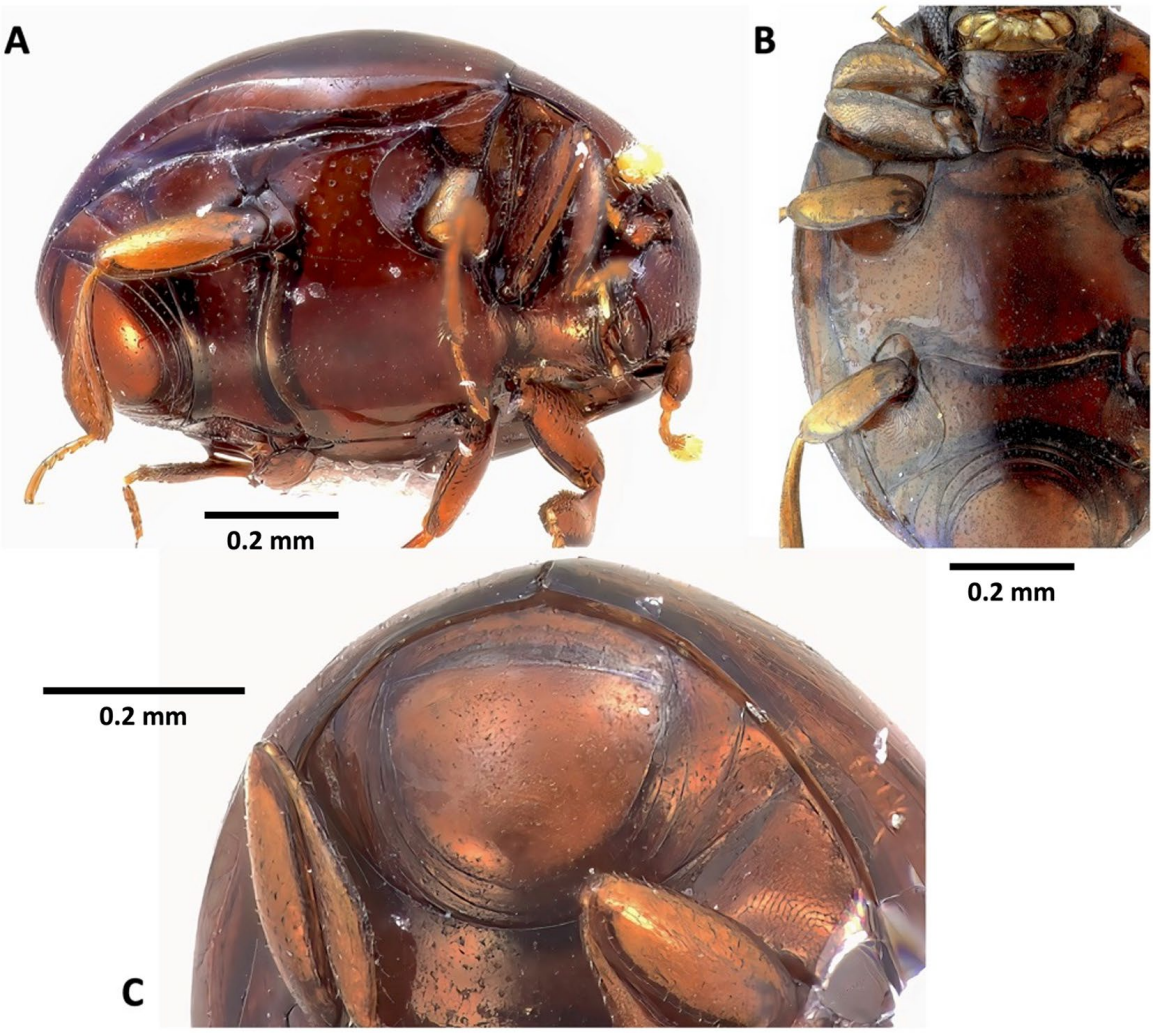
*Bacanius neoponerae* n. sp., habitus ventro-lateral (**A**), and ventral (**B**,**C**) views. Total length = 1 mm; width = 0.77 mm. Photos: N. Degallier.

**Figure 4:**
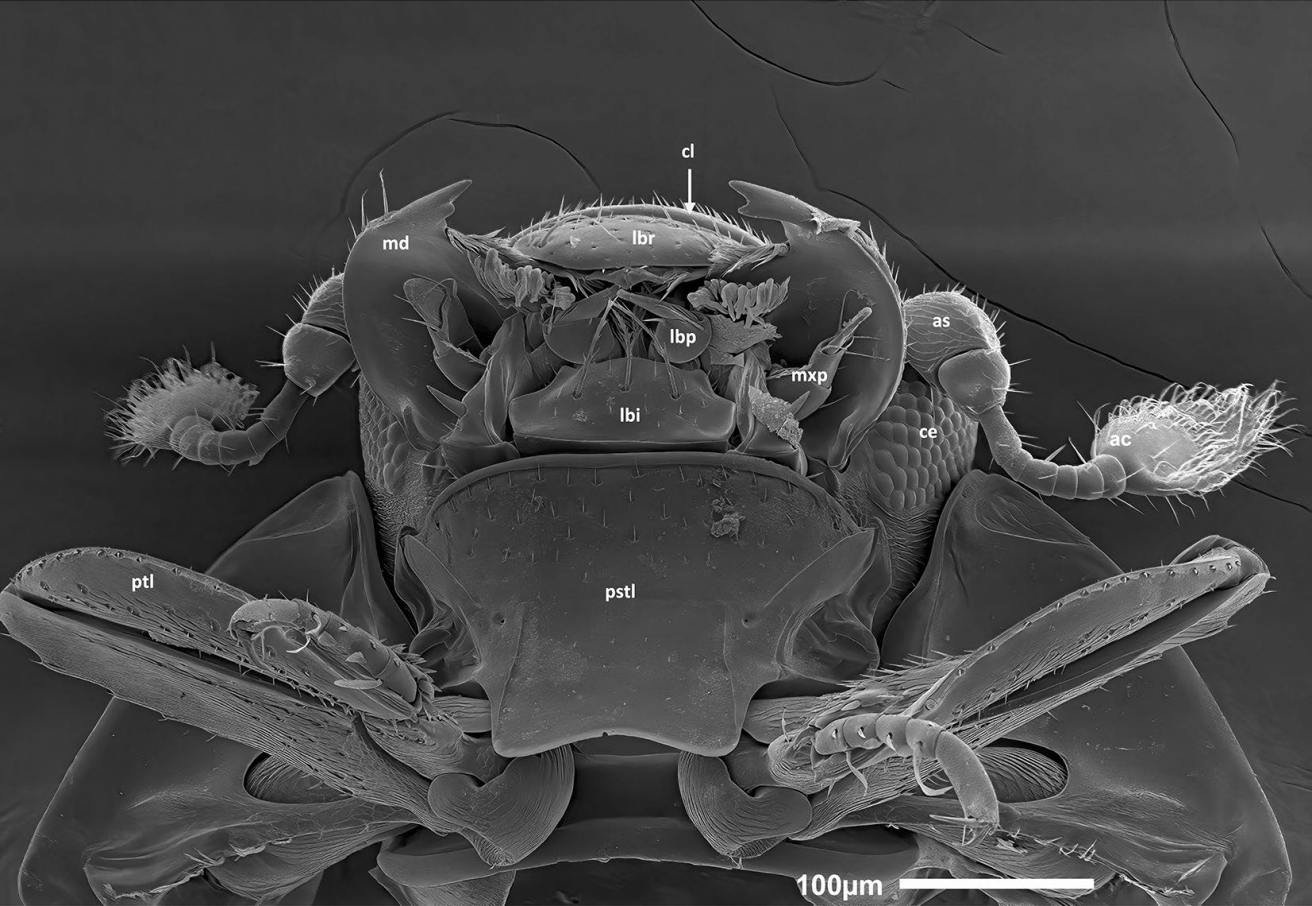
SEM micrograph of the head (fronto-ventral view) of *Bacanius neoponerae* n. sp. showing the mouth parts, the medial part of prosternum, and the prothoracic legs. ac, antennal club; as, antennal scape; ce, compound eye; cl, clypeus; lbi, labium; lbp, labial palp; lbr, labrum; md, mandible; mxp, maxillary palp; pstl, prosternal lobe; ptl, prothoracic legs. Photos: M. Elías-Gutiérrez & G. Pérez-Lachaud.

**Figure 5:**
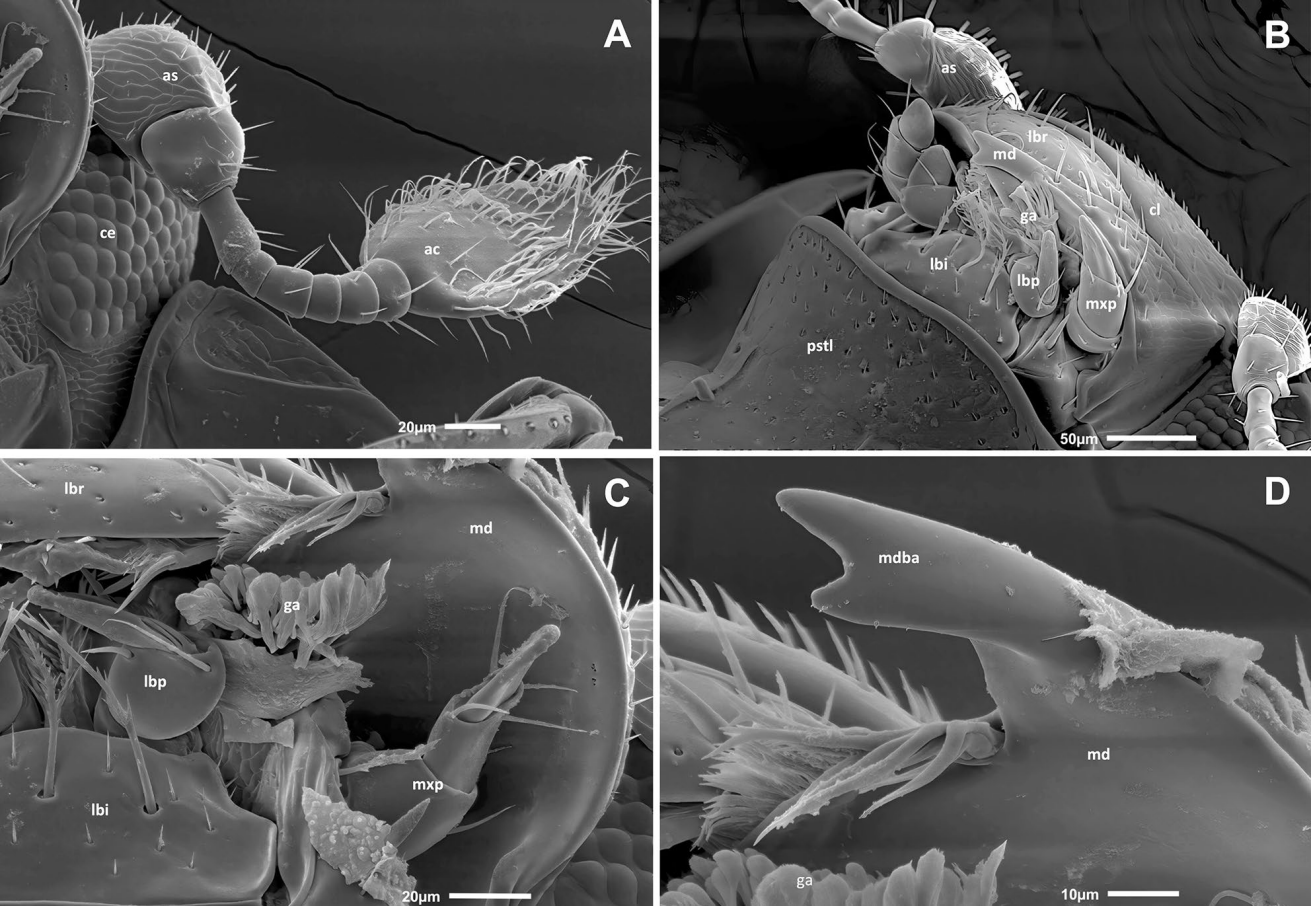
SEM micrographs of *Bacanius neoponerae* n. sp.: left eye and antenna (**A**), mouth parts, prosternal lobe, and setigerous labrum (**B**), mentum and palpi (**C**), left mandible with bifid apex (**D**). ga, galea; mdba, mandible bifid apex; other abbreviations as in Fig. 4. Photos: M. Elías-Gutiérrez & G. Pérez-Lachaud.

**Figure 6:**
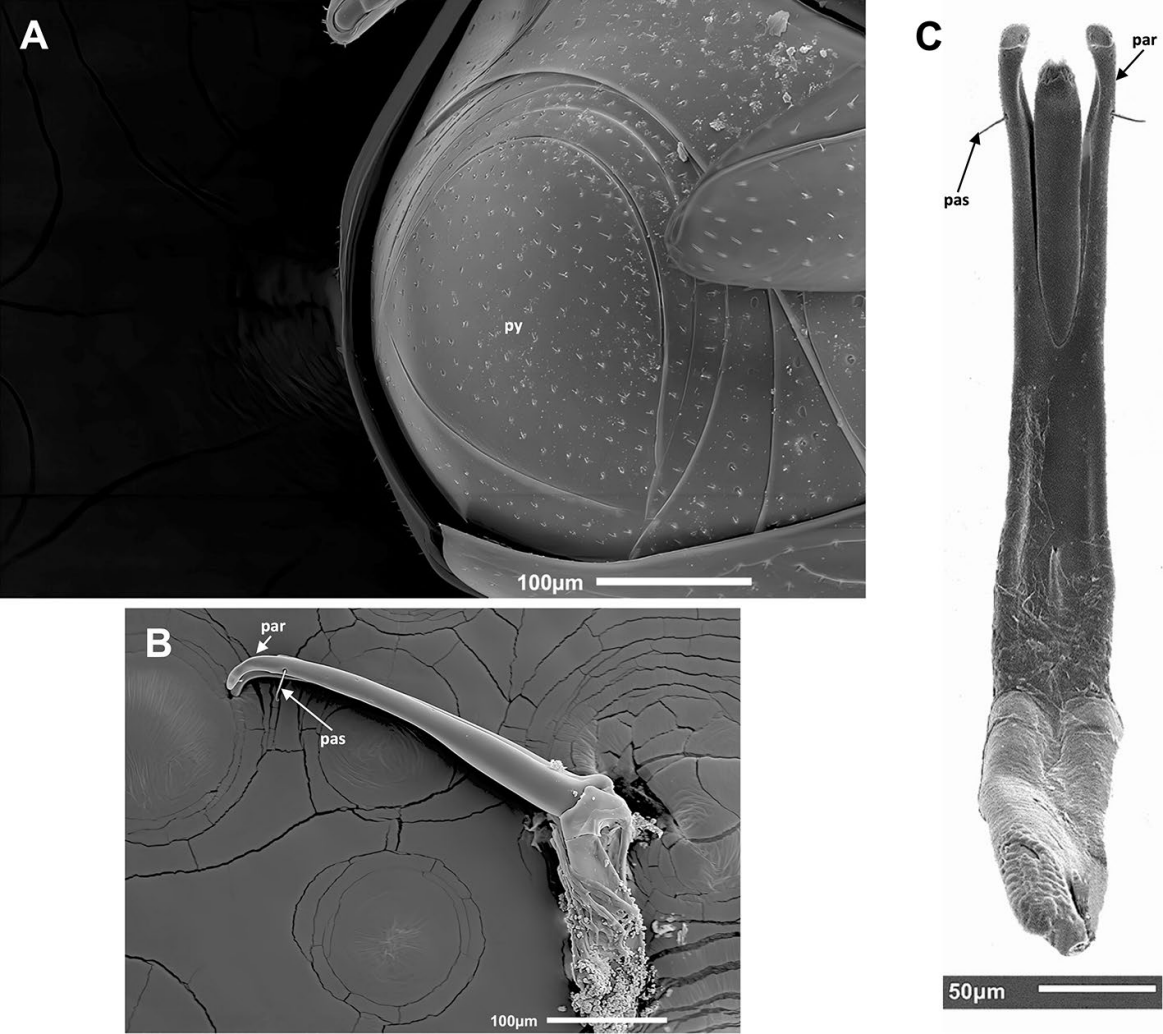
SEM micrographs of *Bacanius neoponerae* n. sp.: pygidium (**A**), aedeagus right lateral view (**B**) and ventral view (**C**). par, paramere of the aedeagus; pas, preapical seta; py, pygidium. Photos: M. Elías-Gutiérrez & G. Pérez-Lachaud.

***Bacanius neoponerae*** Degallier & Gomy, new species [https://zoobank.org/urn:lsid:zoobank.org:act:C618E6B3-C8A5-4CB9-B6D3-BB02674ED4F8]

(Figs. 2, 3, 4, 5 and 6, S1, S2)

**Type locality.** Ejido Blasillo, Campeche, Mexico

HOLOTYPE (sex undetermined, due to the difficulty of safely dissecting such a tiny specimen): Mexico, Campeche, Ejido Blasillo, [Nest 72] 18° 07′ 29.0100″ N, 89° 19′ 53.8121″ W, 261 m asl, 19-III-2018 [MNHN].

PARATYPES: Mexico, Campeche, Ejido Blasillo: 2 ex., [Nest 21] 18°07′14.6172″ N, 89° 19′ 20.4649″ W, 247 m asl, 11-III-2017 [MNHN]; 2 ex., *idem* [FMNH]; 2 ex., *idem* [CHYG]; 2 ex., *idem,* [Nest 26] 18° 07′ 13.6056″ N, 89° 19′ 47.7929″ W, 263 m asl, 10-VI-2017 [CHND]; 1 ex., *idem* [CHYG]; 1 ex., *idem* [Nest 27] 18° 07′ 13.6056″ N, 89° 19′ 47.7929″ W, 263 m asl [ECO-CH-AR, C-2571]; 1 ex., *idem* [CHYG]; 1 ex., *idem*, [Nest 71] 18° 07′ 27.6888″ N, 89° 19′ 52.7064″ W, 261 m asl, 19-III-2018 [CHND]; 1 ex., *idem* [ECO-CH-AR, C-2572]; 2 ex., *idem,* [Nest 72] 18° 07′ 29.0100″ N, 89° 19′ 53.8121″ W, 261 m asl [CHND]; 1 ex., *idem* [ECO-CH-AR, catalog number C-2574]; 1 ex., *idem* [Nest 87] 18° 07′ 15.8448″ N, 89° 19′ 50.8152″ W, 238 m asl, 16-I-2019 [CHYG]; 2 ex., *idem*, 18°07′14.6172″ N, 89° 19′ 20.4649″ W, 247 m asl, 11-III-2017, in material between the bromeliad leaves, outside the ant nests [CHND]; Mexico, Quintana Roo, Sian Ka'an: 1 ex., [Nest 39] 19° 42′ 21.6000″ N, 87° 49′ 46.1191″ W, 12 m asl, 16-VIII-2017 [MNHN]; 2 ex., *idem*, [Nest 43] 19° 42′ 28.3716″ N, 87° 49' 37.2919″ W, 15 m asl [CHND]; 1 ex., *idem* [ECO-CH-AR, C-2570]; 2 ex., *idem* [CHYG]; 2 ex., *idem* [Nest 50] 19° 43′ 10.5492″ N, 87° 48' 43.6449″ W, 24 m asl, 21-X-2017 [BMNH]; 1 ex., *idem* [Nest 52] 19° 42′ 50.8212″ N, 87° 49′ 08.7931″ W, 12 m asl, [CHND]; 3 ex., *idem* [Nest 78] 19° 40′ 41.5560″ N, 87° 51′ 52.9818″ W, 10 m asl, 12-IV-2018 [CHND]; 3 ex., *idem*, in material in the bromeliad leaves, outside the ant nests [CHND]; Mexico, Campeche, Nuevo Becal: 2 ex., [Nest 55] 18° 36′ 39.3552″ N, 89° 16′ 15.5372″ W, 239 m asl, 4-XII-2017 [CHYG]; 1 ex., *idem* [Nest 56] 18° 36′ 16.9956″ N, 89° 16′ 41.4997″ W, 233 m asl [ECO-CH-AR, C-2573]; 1 ex., *idem* [Nest 60] 18° 36′ 32.4864″ N, 89° 16′ 42.9678″ W, 238 m asl, 9-III-2018 [MNHN]; 1 ex., *idem* [Nest 66] 18° 36′ 38.3112″ N, 89° 16′ 34.6558″ W, 231 m asl [CHND]; 1 ex., *idem* [Nest 68] 18° 36′ 38.6676″ N, 89° 16′ 32.2050″ W, 228 m asl [CHND].

*Habitus* (Figs. 2, 3). Length (pronotum + elytra): (0.92–)0.99(–1.11) mm. Maximum width: (0.71–)0.77(–0.85) mm. Maximum thickness: (0.51–)0.58(–0.65) mm, (1.25–)1.29(–1.33) times as long as wide, (1.6–)1.7(–1.86) times as long as thick (N = 12). Body elongated oval, convex dorsally. Dorsal surface, at least on the pronotum, with short setigerous simple punctation.

*Head* (Figs. 2B, 3A, 4, 5A–D). Eyes present. Labrum setigerous. Frons smooth between the punctures. Punctuation of the clypeus equal to or smaller than that of the frons. Apex of mandibles bifid. Antennal scape not dilated. Antennal club without separate sutures. Margins of the forehead above the eyes diverging forward.

*Pronotum* (Figs. 2, S1). Punctuation (diameter about 0.01 mm) not joined by grooves, punctures well marked, 1.5 to 2 diameters apart, coarser on the disk than laterally. Anterior angles of the pronotum with a pore. Base without a row of distinct punctures. Scutellum invisible. Pronotal stria close to the anterior margin (less than 0.018 mm) and not crenulate.

*Elytra* (Figs. 2A, C, S1, S2). Punctuated, without a common prebasal zone punctuated differently on either side of the suture; presence of a pore to the outer third of the base. Punctures not connected by grooves, uniform and smaller than those on the pronotum. Disc without transverse stria. Outer half of the elytra with 2 striae (dorsal and subhumeral/marginal), of which the dorsal and subhumeral are at least twice as distant at the base as apically. Dorsal stria not curved along the base, being shortened basally, reaching the apex but not the sutural angle backwards. Subhumeral stria complete, not united to the dorsal stria apically; elytra without additional basal striae. Sutural stria absent. Punctuation of the apex non-strigate. Epipleura impunctate, epipleural stria present, sinuate and complete. A pore is present on the basal quarter and ventrally to the stria.

*Pygidium* (Figs. 3A, C, 6A, S2). Pygidium less strongly punctuated than elytra, the punctures rounded, 2–4 diameters apart.

*Sterna* (Figs. 3, 4, 5 B, 6A, S2). Prosternal lobe with an anterior marginal stria, slightly emarginated in the middle, punctuated only on its anterior half, without irregular longitudinal striation. Alae of the prosternum incised to receive the antennal funicle. Prosternal striae converging forward and divergent backwards. Prosternal carina punctuated. Mesosternum without striae or sulci, with a transverse line of larger points forward, with the marginal stria laterally in continuity with the lateral metasternal stria. Meso-metasternal disc smooth or finely punctuated (diameter of punctures ≤ 0.009 mm). Meso-metasternal stria absent, the darker trace of the suture visible and curved backwards. Disc of the metasternum completely smooth, without fine apical median keel, sometimes with a low preapical tubercle, without lateral longitudinal rows of punctures, lateral metasternum punctuated, with only a lateral stria and an "S"-shaped short mesopostcoxal stria on each side. Mesopostcoxal area punctuated and mesepimeron punctuated, the latter with a stria forming a very open angle. Metepimeron impunctate. First abdominal ventrite with rounded punctuation, as large or larger than that of the lateral metasternum, without a row of distinct punctures along its anterior margin, with one lateral stria. Postmetacoxal stria absent.

*Legs* (Figs. 2B, 3A, B, 4, S2). Protibiae with 1–2 denticles along the outer edge, punctuated on their ventral side. Protarsi without modified setae in addition to simple ones. Profemorae ventrally with a microstriate area.

*Male genitalia* (Figs. 6B, C). Parameres of the aedeagus curved ventrally at the apex, with two preapical setae ventrally but without apical ones.

**Distribution.** Known only from Mexico.

**Biological information.** Specimens of the new species were found associated with *N. villosa* ants nesting in *A. bracteata* bromeliads. In addition, two specimens were found in a colony of another ant species, *C. atriceps*, also inhabiting *A. bracteata*.

**Etymology.** The name relates to the host of the new species, belonging to the ponerine ant genus *Neoponera*.

**Remarks**. To our knowledge, *B. neoponerae* is part of the Neotropical Bacaniini (Dendrophilinae) characterized by a length (pronotum + elytra) greater than 0.9 mm, with invisible scutellum, no prescutellar stria at the base of the pronotum, the anterior stria of the pronotum close to the margin and not crenulate, the first dorsal stria of the elytra shortened forward and reaching the sutural angle apically, no sutural or transverse elytral striae, and the pronotal punctuation not joined by furrows. According to Wenzel's key^31^, the Neotropical species that most resemble *B*. *neoponerae* are *B. convergens* Schmidt, described from southeastern Brazil, and *B. subcarinatus* from Veracruz, Mexico. *Bacanius neoponerae* is distinguished from these species by the following combination of characters: prosternal lobe punctuated, without longitudinal striation (with irregular longitudinal striation in *B. convergens*); short setigerous punctuation (glabrous in *B. convergens*); punctuation of the clypeus equal to or smaller than that of the forehead (larger in *B. convergens*); visible meso-metasternal suture (invisible in *B. convergens*); elytra with a pore situated at the outer third of the base (at the middle of the elytral base in *B. subcarinatus*); elytral punctuation uniform (stronger along the suture in *B. subcarinatus*); dorsal stria of elytra shortened at the base (complete or barely shortened in *B. subcarinatus*); pygidium less strongly punctuated than the elytra (more strongly punctuated than the elytra in *B. subcarinatus*).

### Molecular analyses

**Figure 7:**
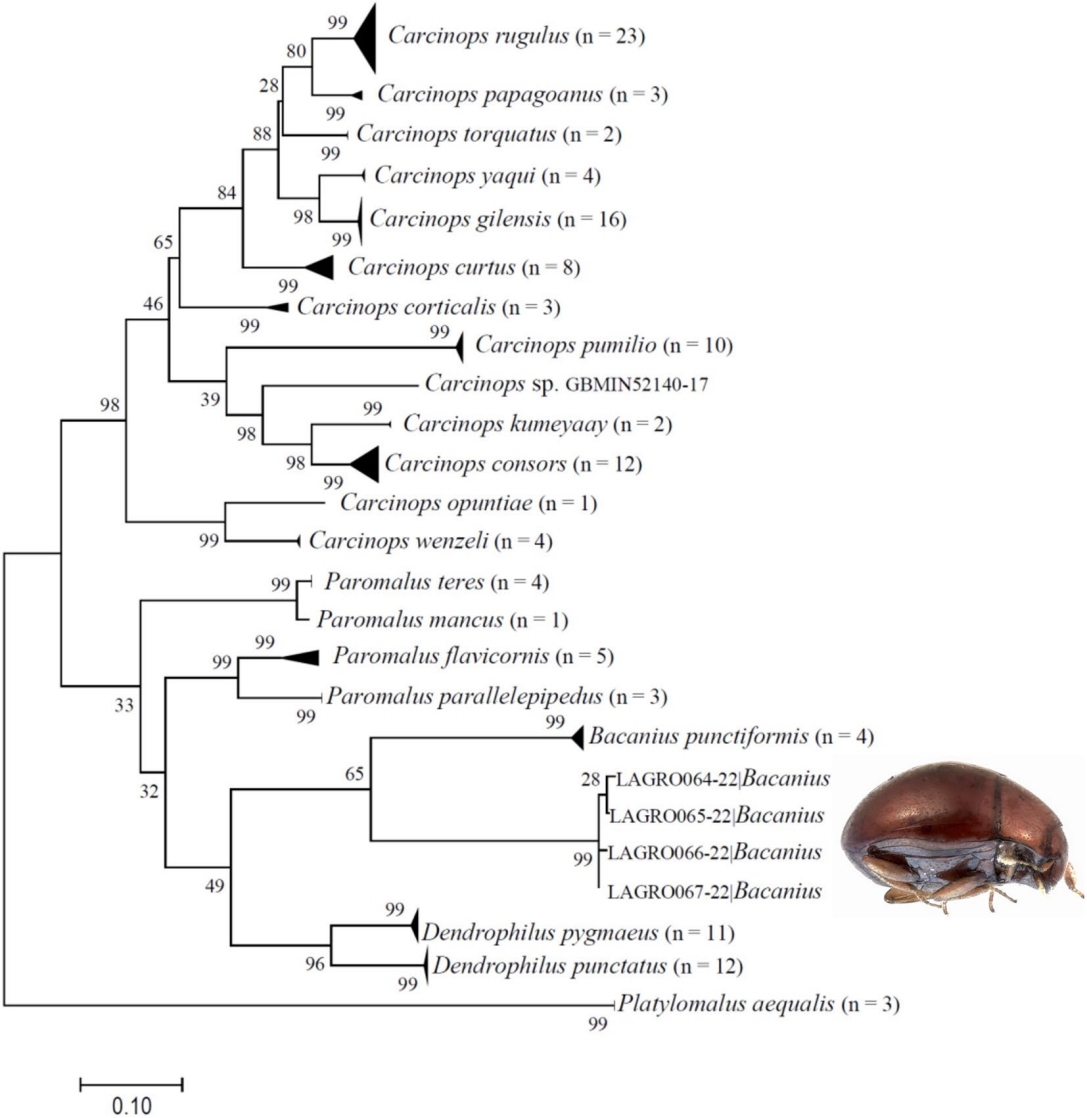
Condensed ID-Tree. The evolutionary history was inferred by using the Maximum Likelihood method based on the Tamura-Nei^53^ model (n = number of sequences in each branch).

Out of the five individuals extracted, four yielded CO1 fragment sequences with a length of 600 bp. The maximum likelihood phylogenetic analysis placed *B*. *neoponerae* in a clade containing the only other *Bacanius* species for which COI sequences have been published (*B. punctiformis*) but clearly separated from this species and from others in the same subfamily (Fig. 7) confirming its status based on morphology. DNA barcoding supported the generic placement of the new species. Pairwise sequence distances between the new species and *B. punctiformis* ranged from 2.43 to 2.49%. No genetic variability was found in COI sequences. The four sequences obtained here were grouped in a separate clade and they are similar, with pairwise differences from 0.02 to 0.1%.

### Ecology

A total of 173 specimens of *B. neoponerae*, all adults, were retrieved from the epiphytic bromeliad *A. bracteata* sheltering *N. villosa* colonies. Globally, histerids were associated with 43.6% (34/78) of the colonies: 10 of the colonies sampled in Sian Ka’an, 8 from Nuevo Becal, and 16 from Ejido Blasillo. Ninety-seven specimens (56.1%), observed in more than two thirds of these colonies (24/34) (range: 1–18 individuals per colony), were found in the central part of the bromeliads where the ant nests were established, mainly in the detritus inside the nest chambers. Seventy-six additional specimens (43.9%) were also found in the bromeliads inhabited by *N. villosa*, but outside the ant nest, among the detritus accumulated between the peripheral leaves of *A. bracteata*. Wherever the location of the histerid beetles within the bromeliad, no direct interaction between them and the ants or their brood could be observed.

The Random Forest classification tree showed that the most important variables predicting the association of *B. neoponerae* with the *N. villosa* colonies were altitude of the sampling site, followed by colony size, monthly rainfall and collecting zone in decreasing order of importance (Fig. S3). Histerids were associated with ant colonies year-round. Thirty-eight percent of the colonies collected during the dry season, 42% of those collected during the rainy season, and 52% of those collected during the cold season harbored histerids. However, quantitatively, half the histerids were recovered during the dry season. During this season, colonies associated with histerids contained a higher mean number of specimens (7.73 ± 2.5 (n = 12) than during the rainy and cold seasons (5.2 ± 1.8 (n = 8) and 3.3 ± 0.5 (n = 14), respectively) (Fig. S4); however, figures varied widely and differences in abundance were non-significant (Kruskal–Wallis test, H = 0.04, n.s.). At the spatial scale, colonies collected at Ejido Blasillo (i.e., at a relatively higher altitude) were more frequently encountered in association with histerids and the beetles were more numerous. A total of 90 individuals were collected at Ejido Blasillo, 64 in Sian Ka’an and 19 at Nuevo Becal from 29, 25, and 24 colonies, respectively. In addition, larger colonies favored the presence of *B. neoponerae*: the mean colony size of the colonies that were associated with histerids was significantly higher (227 ± 27 adult ants), than that of the colonies without histerids (173 ± 15 adult ants) (Student t test, *p* = 0.034). The colony size frequency distribution of both colonies sheltering histerids and those without beetles followed a normal distribution (W_with_ = 0.902, *p* < 0.05; W_without_ = 0.468, *p* < 0.05).

## Discussion

Histeridae are robust beetles characterized, in general, by a broadly globular body shape and protective morphological structures complemented by the ability to retract the head and appendages into anatomical cavities, which further protect against attack^5,16,57–59^. According to Parker and Kronauer^60^, such a protective anatomical ground plan of histerids and some other beetles such as aleocharine staphylinids, predisposed these beetles to myrmecophily. Many clown beetle species, primarily in the subfamilies Haeteriinae and Chlamydopsinae, are known to be associated with ants in diverse ways and several are entirely ant-dependent, displaying complex strategies to integrate the host nests and cope with worker aggressiveness^16,59,61^. Less is known, however, about myrmecophilous species belonging to other subfamilies, although associations with ants have been reported on various occasions^13,62^. Species of the tribe Bacaniini (Dendrophilinae) have rarely been suspected of being associated with ants. A species of the genus *Australanius*, *A. verrucosus* Gomy, was described from material preserved along with two ant workers^63^ of the dolichoderine genus *Iridomyrmex* (determination Georg Fischer, pers. comm., and J-PL), and there is only one previous report of an unidentified species of *Bacanius* recorded with the army ant *Eciton dulcium crassinode* (Dorylinae)^33^. However, the nature of the latter association was not clearly determined and may be facultative since only one specimen (a male) was collected from a refuse deposit in a bivouac and the species may casually exploit resources there, as has been reported for species in six histerid subfamilies (Abraeinae, Dendrophilinae, Haeteriinae, Histerinae, Saprininae, and Tribalinae) collected in the refuse piles of various ant genera such as *Atta*, *Acromyrmex*, *Eciton*, *Pheidole*, and *Solenopsis*^24,25,33,62^.

Here we describe a new species of the tribe Bacaniini and provide ecological information on its association with the arboreal ant *N. villosa*. Ecological information on histerid beetles is rather scarce and biological information on *Bacanius* species almost absent. Current records include subcortical tree samples and leaf litter samples, including for the type species *B. tantillus* LeConte, which was described from material collected from under bark and in fungi^64^. According to Kovarik^65^, the ancestral feeding habits of most adult histerids is predation; however, adults of several lineages feed on fungal spores or microbiota coatings, and Pražák^21^ mentions members of the genus *Bacanius* as spore-feeding species, but without giving any precise reference. Most spore-feeding taxa are also capable of some predation, and their larvae are predatory as in other histerid beetles^65^, but specialized species have modified maxillary galea with setae that serve as combs for gathering spores^65^. Interestingly, the reproductive rate of these species is highest under conditions that are also ideal for fungal fructification (heat, humidity)^21^ such as those found within the bromeliads. Histerids also appear to be preadapted to exploiting ephemeral systems: there are only two larval instars, and their large eggs produce precocious larvae capable of capturing prey shortly after hatching^65^. Future examination of the mouth parts and gut contents of *B. neoponerae* adults may provide information on the feeding habits of the new species.

At regional and local scales, we found that *B. neoponerae* was associated with 43.6% of the colonies of *N. villosa* collected in *A. bracteata* and, in more than two-thirds of these, specimens were located within the brood chambers conditioned by the ants in the core of the bromeliads, strongly suggesting a myrmecophilic syndrome. In the Yucatan Peninsula, the new species was also found in one of the two *A. bracteata* bromeliads inhabited by another ant species belonging to a different ant subfamily (*C. atriceps*, Formicinae), although in very small numbers (only two specimens), suggesting a close but not exclusive association with *N. villosa* ants and, presumably, a strong preference for microhabitats created by litter accumulation and ant activities in the tank bromeliad.

In addition to its small size (about 1 mm), *B. neoponerae* is protected from attack by its globular body shape, thick cuticle, and ability to fully withdraw the first pair of legs and the head. Unspecialized, unwelcome ant guests are known to use simple behavioral strategies to escape host detection, such as prudent behavior^66^. Besides slow movement, small size seems to contribute to evade ant aggression: myrmecophiles much smaller than their host are, in general, mostly ignored while those that match the host’s size are attacked^59^. As shown in a previous study, most arthropod species associated with *N. villosa* established in *A. bracteata* are small body size arthropods relative to their host (workers are 12–13 mm), mainly Coleoptera (various species of Staphylinidae, Ptiliidae, Nitidulidae) and Hymenoptera (other small ant species or small parasitoid wasps)^35^. Small body size and prudent behavior in addition to a protective body shape, may favor cohabitation of *B. neoponerae* with this aggressive ant within bromeliads. The fact that the beetles were found inside the nests suggests that ants tolerate or ignore these small beetles. Their aggressive hosts may indirectly provide the beetles a degree of protection from predators as well as many resources and the benefit of a warm and humid environment.

Based on our analyses, a combination of factors determined the presence of *B. neoponerae* in our samples: they were more frequently encountered in colonies at relatively higher altitude and during the driest months of the year and were particularly abundant in association with larger colonies of *N. villosa*, the last two drivers being determinant for beetle presence. Tank bromeliads are unique canopy microhabitats where species-specific aquatic and terrestrial biota develop. Like other tank bromeliads, *A. bracteata* provides refuge for a diverse array of invertebrates and vertebrates, particularly during the dry season when they account for a substantial portion of the water available in the canopy^35,67–70^. Tank bromeliads can store from a few milliliters to several liters of water, depending on the size and number of reservoirs per plant^71^. In *A. bracteata*, water reservoirs are formed by the tightly interlocked leaves and contain 34 ml on average^71^, and groups of shoots at different ontogenetic stages are commonly present on a given tree. In experimental settings, *N. villosa* colonies tend to select the larger plant when offered two *A. bracteata* plants of different sizes^43^, therefore populous colonies in the field are likely to occupy and defend larger plants or groups of plants with the largest water reservoirs thereby exerting greater attraction to other arthropods. Furthermore, the larger the ant colonies are, the more substantially they can alter their nesting sites by creating different microhabitats, thus increasing the availability of resources for potential guests. In fact, the most populous ant species are thought to support a highly diverse symbiont community due to the different microhabitats present in their nests and the longevity of the colonies, that allow to sustain numerous associates over longer periods^2,72^. The correlation we found in our study between *B. neoponerae* abundance and *N. villosa* colony size supports this hypothesis.

Our report is the second case of a *Bacanius* associated with ants and the first case of a Dendrophilinae associated with ponerine ants. This is also only the fourth time a histerid species has been found cohabiting with ants of the subfamily Ponerinae. An adult of *Acritus megaponerae* Bickhardt (Abraeinae) has previously been described from Bulawayo, Zimbabwe, in a nest of *Megaponera anali*s (Latreille) (= *M. foetens*)^73^, and a single specimen of *Mecistostethus loretoensis* (Bruch) (= *Tarsilister loretoensis*) (Histerinae) has been reported in Argentina in the brood chamber of *Pachycondyla striata* Smith^74^; finally, unidentified adult histerids, probably scavengers, have been found alive near the refuse chambers, inside several nests of the giant ant *Dinoponera grandis* (Guérin-Méneville) (= *D. australis*) in Brazil^75^. Notably, in all these cases the host is distinguished by its high aggressiveness and corresponds to a large ant species relative to the histerid guest (12–13 mm vs*.* about 1 mm in the association with *N. villosa*; about 13 mm^76^ vs*.* about 3 mm^74^ in the association with *P. striata*; 5–18 mm^77^ vs. about 0.8 mm^73^ in the association with *M. analis*; and *D. grandis*, with a body length of 22–27 mm^78^, is known as one of the largest ant species). Much more research needs to be carried out in the future to reveal whether this new species is restricted to ants nesting in *A. bracteata* and to determine whether *B. neoponerae* is an obligate or facultative guest of *N. villosa* or a generalist commensal of hosts sharing the same nesting habitat. Specifically, a close examination of other ant colonies found in *A. bracteata*, including the dolichoderine ant *Dolichoderus bispinosus* Olivier, the main competitor of *N. villosa* in the Yucatan Peninsula in using this tank bromeliad as nesting site^34,79^, would clarify whether *B. neoponerae* is primarily dependent on *N. villosa* or on specific microhabitats within this tank bromeliad.

### Supplementary Information

Supplementary Information.

## Data Availability

All data generated or analyzed during this study are included in this published article and its supplementary information files.
