# Supplementary material for: Cohabitation with aggressive hosts: description of a new microhisterid species in nests of a ponerine ant with ecological notes
Source: Sci Rep. 2023 Oct 28;13:18484. doi: 10.1038/s41598-023-45692-1 (PMC10613264; doi:10.1038/s41598-023-45692-1)
Supplement: Supplementary file 1 — Supplementary Information. [file 41598_2023_45692_MOESM1_ESM.pdf]

Supplementary Information

Cohabitation with aggressive hosts: Description of a new microhisterid species in nests of a ponerine ant with ecological notes

Gabriela Pérez-Lachaud[1*], Nicolas Degallier[2], Yves Gomy[3], Manuel Elías-Gutiérrez[4], Franklin H. Rocha[1,5], Jean-Paul Lachaud[1*]

[1]El Colegio de la Frontera Sur, Departamento de Conservación de la Biodiversidad, Avenida Centenario Km 5.5, Chetumal, Mexico.
[2]120 rue de Charonne, 75011 Paris France.
[3]2 boulevard Victor Hugo, 58000 Nevers France.
[4]El Colegio de la Frontera Sur, Departamento de Ecología y Sistemática Acuática, Avenida Centenario Km 5.5, Chetumal, Mexico.
[5]Present address: Dpto. Apicultura, Campus de Ciencias Biológicas y Agropecuarias, Universidad Autónoma de Yucatán, Mérida, Yucatán, México.

*Authors for correspondence: jlachaud@ecosur.mx; igperez@ecosur.mx

Content

French extensive summary.

Table S1. Specimen information. Dendrophilinae COI sequences were retrieved from Genbank and Bold Systems databases.

Figure S1. SEM micrographs of the dorsal view of *Bacanius neoponerae* n. sp. (A) with details of the punctures present on the pronotum and the elytra (B-D). po: pore; pr: pronotum; ely: elytra. (Photos: M. Elías-Gutiérrez & G. Pérez-Lachaud).

Figure S2. SEM micrograph of the ventro-lateral view of *Bacanius neoponerae* n. sp. abd1: abdominal ventrite 1; dst: elytral dorsal stria; epi: epimeron with one epimeral stria; epst: epipleural stria; mesost: mesosternum; metast: metasternum; po: pore; pstc: prosternal carina; pstl: prosternal lobe; py: pygidium; shst: elytral subhumeral stria. (Photos: M. Elías-Gutiérrez & G. Pérez-Lachaud).

Figure S3. Drivers of *Bacanius* presence in *N. villosa* colonies. The classification tree shows how the data are distributed according to the most important variables predicting the association of *B. neoponerae* with *N. villosa* colonies (altitude of the sampling site, colony size, monthly rainfall and collecting zone). The response variable is binary (presence/absence) with 0.44 of ant colonies hosting the beetle (relative proportion 100%). Squares indicate the relative proportion of data belonging to each group (i.e., the number of observations in a group). The values of the explanatory variables used for the partition data are indicated below the squares. Zone 1: Sian Ka'an; Zone 2: Nuevo Becal; Zone 3: Ejido Blasillo.

Figure S4. Boxplot of the number of *Bacanius neoponerae* found in *N. villosa* colonies nesting in *Aechmea bracteata* across different seasons (dry, rainy, cold) in southeastern Quintana Roo, Mexico (n = 12, 8, 14 nests, respectively). Each box shows upper and lower quartiles along with maximum and minimum (whiskers), median (thick line) and outliers.

Résumé extensif. Une nouvelle espèce de micro-histéride, *Bacanius neoponerae*, est décrite à partir des fourmilières de la fourmi arboricole *Neoponera villosa* établies dans les broméliacées épiphytes *Aechmea bracteata*, dans la Péninsule du Yucatan au Mexique. La nouvelle espèce se distingue des autres Bacaniini néotropicaux (Dendrophilinae) par la combinaison de caractères suivante: longueur moyenne (pronotum + élytres) supérieure à 0,9 mm, ponctuation du corps brièvement sétigère, ponctuation du clypeus égale ou plus fine que celle du front, scutellum invisible, pas de strie pré-scutellaire à la base du pronotum, strie marginale antérieure du pronotum près du bord et non crénelée, ponctuation pronotale non reliée par des sillons, pore basal des élytres situé au tiers externe de la base, ponctuation élytrale uniforme, première strie dorsale des élytres raccourcie en avant et atteignant l'angle sutural apical, pas de stries élytrales suturales ou transversales, pygidium moins fortement ponctué que les élytres, lobe prosternal ponctué et sans striation longitudinale. Les coléoptères adultes ont été trouvés dans les chambres à couvain ou les tas d'ordures à l'intérieur des nids, mais aussi dans la matière organique en décomposition entre les feuilles des broméliacées, à l'extérieur du nid. Aucune interaction directe entre les fourmis et les micro-histérides n'a pu être observée mais plusieurs éléments suggèrent une relation étroite soit avec les fourmis, soit avec un micro-habitat spécifique à l'intérieur des nids de fourmis ou des broméliacées. Une analyse écologique a montré que l'altitude du site d'échantillonnage, la pluviométrie et la taille des colonies de *N. villosa* étaient les principales variables favorisant l'association entre ces coléoptères et les fourmilières. Près de la moitié des colonies de *N. villosa* sont associées à *B. neoponerae*, et les colonies plus grandes favorisent leur présence, en particulier pendant les mois les plus secs de l'année. Deux spécimens ont été trouvés dans un nid d'une autre espèce de fourmis, *Camponotus atriceps*, nidifiant également dans *A. bracteata*. *Bacanius neoponerae* n. sp. est la septième espèce du genre *Bacanius* signalée au Mexique. C'est la deuxième fois qu'une espèce du genre *Bacanius* est associée à des fourmis et seulement la quatrième mention d'un coléoptère histéride cohabitant avec des fourmis ponérines qui, dans tous les cas, présentent une grande différence de taille par rapport à leurs hôtes. La petite taille de ces coléoptères et leur structure corporelle très compacte peuvent faciliter leur cohabitation avec des hôtes aussi agressifs. De nombreuses autres recherches devront être menées à l'avenir pour révéler si cette nouvelle espèce est limitée aux fourmis nichant dans *A. bracteata* et pour déterminer si *B. neoponerae* est un hôte obligatoire ou facultatif de *N. villosa* ou un commensal généraliste d'hôtes partageant le même habitat de nidification. En particulier, un examen approfondi des autres colonies de fourmis trouvées dans *A. bracteata*, notamment la fourmi dolichoderine *Dolichoderus bispinosus*, le principal concurrent de *N. villosa* dans la péninsule du Yucatan dans l'utilisation de cette broméliacée comme site de nidification, permettrait de clarifier si *B. neoponerae* est principalement dépendante de *N. villosa* ou de microhabitats spécifiques au sein de la broméliacée.

Table S1. Specimen information. Dendrophilinae COI sequences were retrieved from Genbank and Bold Systems databases.

Species	Tribe	GenBank accession number	Bold Systems record ID
Bacanius punctiformis	Bacaniini		MPCAN1805-19
Bacanius punctiformis	Bacaniini		MPCAN1806-19
Bacanius punctiformis	Bacaniini		MPCAN688-17
Bacanius punctiformis	Bacaniini		OJIBW334-19
Bacanius n. sp.	Bacaniini	OQ706398	LAGRO064-22
Bacanius n. sp.	Bacaniini	OQ706396	LAGRO065-22
Bacanius n. sp.	Bacaniini	OQ706397	LAGRO066-22
Bacanius n. sp.	Bacaniini	OQ706395	LAGRO067-22
Dendrophilus punctatus	Dendrophilini	KM443269	
Dendrophilus punctatus	Dendrophilini	MZ633277	
Dendrophilus punctatus	Dendrophilini	MZ633871	
Dendrophilus punctatus	Dendrophilini	HQ954522	
Dendrophilus punctatus	Dendrophilini	KM449998	
Dendrophilus punctatus	Dendrophilini	KU909320	
Dendrophilus punctatus	Dendrophilini	KU910429	
Dendrophilus punctatus	Dendrophilini	KU910472	
Dendrophilus punctatus	Dendrophilini	KU912848	
Dendrophilus punctatus	Dendrophilini	KU913956	
Dendrophilus punctatus	Dendrophilini	KU915066	
Dendrophilus punctatus	Dendrophilini	KU918353	
Dendrophilus pygmaeus	Dendrophilini	HQ559231	
Dendrophilus pygmaeus	Dendrophilini	KM439226	
Dendrophilus pygmaeus	Dendrophilini	KM440850	
Dendrophilus pygmaeus	Dendrophilini	KM443097	
Dendrophilus pygmaeus	Dendrophilini	KM446253	
Dendrophilus pygmaeus	Dendrophilini	KU910753	
Dendrophilus pygmaeus	Dendrophilini	KM441773	
Dendrophilus pygmaeus	Dendrophilini	KM441987	
Dendrophilus pygmaeus	Dendrophilini	KU911724	
Dendrophilus pygmaeus	Dendrophilini	KU917581	
Dendrophilus pygmaeus	Dendrophilini	MZ631586	
Carcinops consors	Paromalini	JX879953	
Carcinops consors	Paromalini	JX879954	
Carcinops consors	Paromalini	JX879955	
Carcinops consors	Paromalini	KX603701	
Carcinops consors	Paromalini	KX603702	
Carcinops consors	Paromalini	KX603703	
Carcinops consors	Paromalini	KX603704	

Carcinops consors	Paromalini	KX603705	
Carcinops consors	Paromalini	KX603706	
Carcinops consors	Paromalini	KX603707	
Carcinops consors	Paromalini	KX603708	
Carcinops consors	Paromalini	KX603709	
Carcinops corticalis	Paromalini	KX603710	
Carcinops corticalis	Paromalini	KX603711	
Carcinops corticalis	Paromalini	KX603712	
Carcinops curtus	Paromalini	KX603716	
Carcinops curtus	Paromalini	KX603713	
Carcinops curtus	Paromalini	KX603714	
Carcinops curtus	Paromalini	KX603715	
Carcinops curtus	Paromalini	KX603717	
Carcinops curtus	Paromalini	KX603718	
Carcinops gilensis	Paromalini	JX880020	
Carcinops gilensis	Paromalini	KX603719	
Carcinops gilensis	Paromalini	KX603730	
Carcinops gilensis	Paromalini	JX879988	
Carcinops gilensis	Paromalini	JX879990	
Carcinops gilensis	Paromalini	JX879994	
Carcinops gilensis	Paromalini	JX879996	
Carcinops gilensis	Paromalini	JX879998	
Carcinops gilensis	Paromalini	JX880004	
Carcinops gilensis	Paromalini	JX880008	
Carcinops gilensis	Paromalini	JX880010	
Carcinops gilensis	Paromalini	JX880012	
Carcinops gilensis	Paromalini	JX880021	
Carcinops gilensis	Paromalini	KX603720	
Carcinops gilensis	Paromalini	KX603727	
Carcinops gilensis	Paromalini	KX603733	
Carcinops kumeyaay	Paromalini	KX603734	
Carcinops kumeyaay	Paromalini	KX603735	
Carcinops opuntiae	Paromalini	KX603736	
Carcinops papagoanus	Paromalini	KX603739	
Carcinops papagoanus	Paromalini	KX603740	
Carcinops papagoanus	Paromalini	KX603741	
Carcinops pumilio	Paromalini	KJ963425	
Carcinops pumilio	Paromalini	KJ965069	
Carcinops pumilio	Paromalini	KJ967216	
Carcinops pumilio	Paromalini	KM447703	
Carcinops pumilio	Paromalini	KM444098	
Carcinops pumilio	Paromalini	KM448124	
Carcinops pumilio	Paromalini	KR905933	
Carcinops pumilio	Paromalini	KU915481	

Carcinops pumilio	Paromalini	KX603761	
Carcinops rugulus	Paromalini	KX603742	
Carcinops rugulus	Paromalini	KX603750	
Carcinops rugulus	Paromalini	KX603743	
Carcinops rugulus	Paromalini	KX603744	
Carcinops rugulus	Paromalini	KX603745	
Carcinops rugulus	Paromalini	KX603746	
Carcinops rugulus	Paromalini	KX603747	
Carcinops rugulus	Paromalini	KX603748	
Carcinops rugulus	Paromalini	KX603749	
Carcinops sp.	Paromalini	KX603762	
Carcinops sp.	Paromalini	JX879957	
Carcinops sp.	Paromalini	JX879958	
Carcinops sp.	Paromalini	JX879959	
Carcinops sp.	Paromalini	JX879960	
Carcinops sp.	Paromalini	JX879961	
Carcinops sp.	Paromalini	JX879962	
Carcinops sp.	Paromalini	JX879963	
Carcinops sp.	Paromalini	JX879964	
Carcinops sp.	Paromalini	JX879965	
Carcinops sp.	Paromalini	JX879966	
Carcinops sp.	Paromalini	JX879967	
Carcinops sp.	Paromalini	JX879968	
Carcinops sp.	Paromalini	JX879969	
Carcinops sp.	Paromalini	JX879970	
Carcinops sp.	Paromalini	JX879971	
Carcinops sp.	Paromalini	JX879972	
Carcinops torquatus	Paromalini	KX603751	
Carcinops torquatus	Paromalini	KX603752	
Carcinops wenzeli	Paromalini	KX603753	
Carcinops wenzeli	Paromalini	KX603754	
Carcinops wenzeli	Paromalini	KX603755	
Carcinops wenzeli	Paromalini	KX603756	
Carcinops yaqui	Paromalini	KX603757	
Carcinops yaqui	Paromalini	KX603759	
Carcinops yaqui	Paromalini	KX603758	
Carcinops yaqui	Paromalini	KX603760	
Paromalus difficilis	Paromalini	KX639724	
Paromalus flavicornis	Paromalini	KM439310	
Paromalus flavicornis	Paromalini	KM445012	
Paromalus flavicornis	Paromalini	KU917631	
Paromalus flavicornis	Paromalini	JF889501	
Paromalus flavicornis	Paromalini	JN299243	
Paromalus mancus	Paromalini	KU875783	

Paromalus parallelepipedus	Paromalini	HQ953480	
Paromalus parallelepipedus	Paromalini	KM444552	
Paromalus parallelepipedus	Paromalini	KM451747	
Paromalus teres	Paromalini		MPCAN232-17
Paromalus teres	Paromalini		MPCAN091-17
Paromalus teres	Paromalini		CNCCG762-12
Paromalus teres	Paromalini		MPCAN090-17
Platylomalus aequalis	Paromalini		MPCAN015-17
Platylomalus aequalis	Paromalini		MPCAN016-17
Platylomalus aequalis	Paromalini		MPCAN017-17

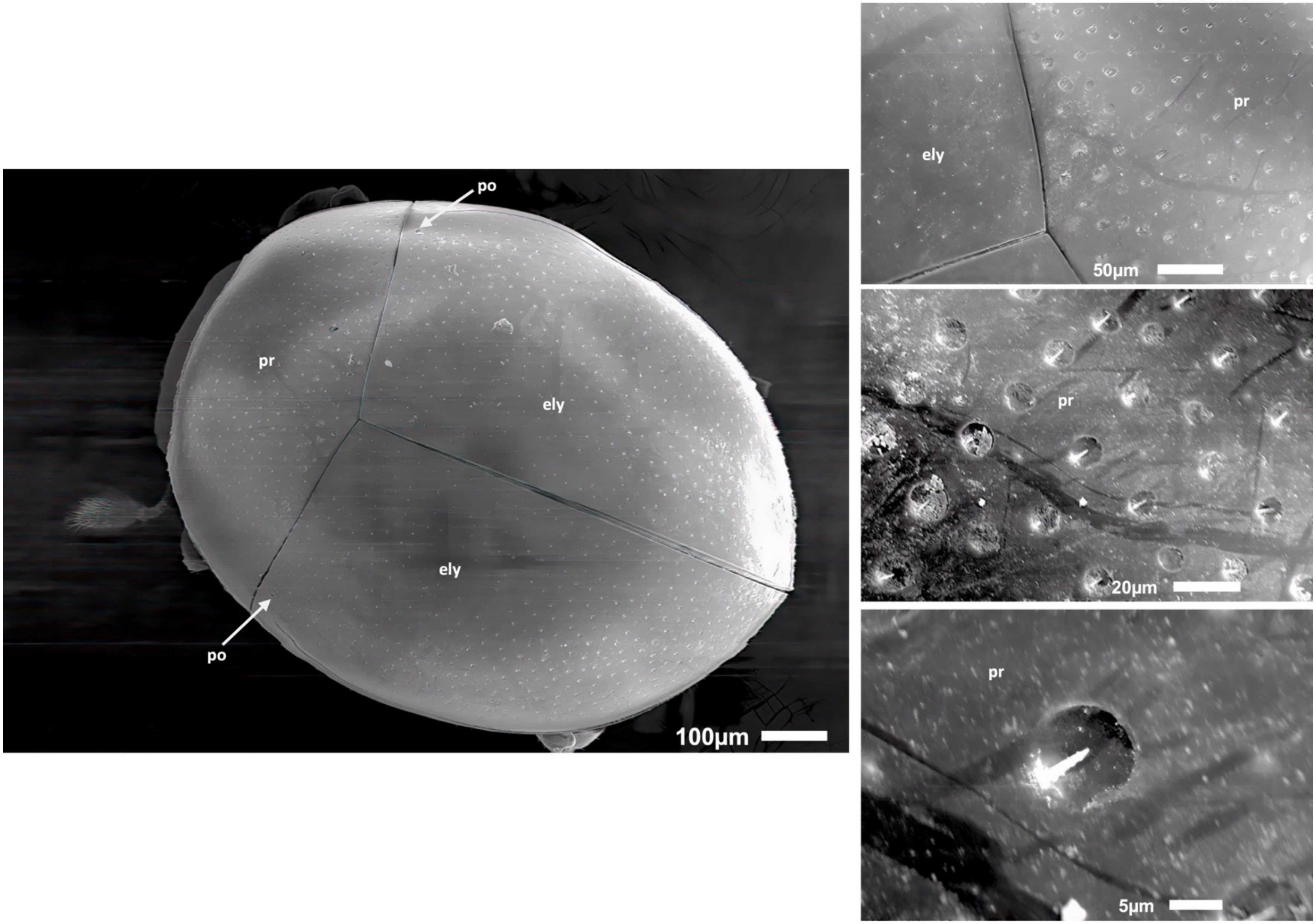

Figure S1. SEM micrographs of the dorsal view of *Bacanius neoponerae* n. sp. (A) with details of the punctures present on the pronotum and the elytra (B-D). po: pore; pr: pronotum; ely: elytra. (Photos: M. Elías-Gutiérrez & G. Pérez-Lachaud).

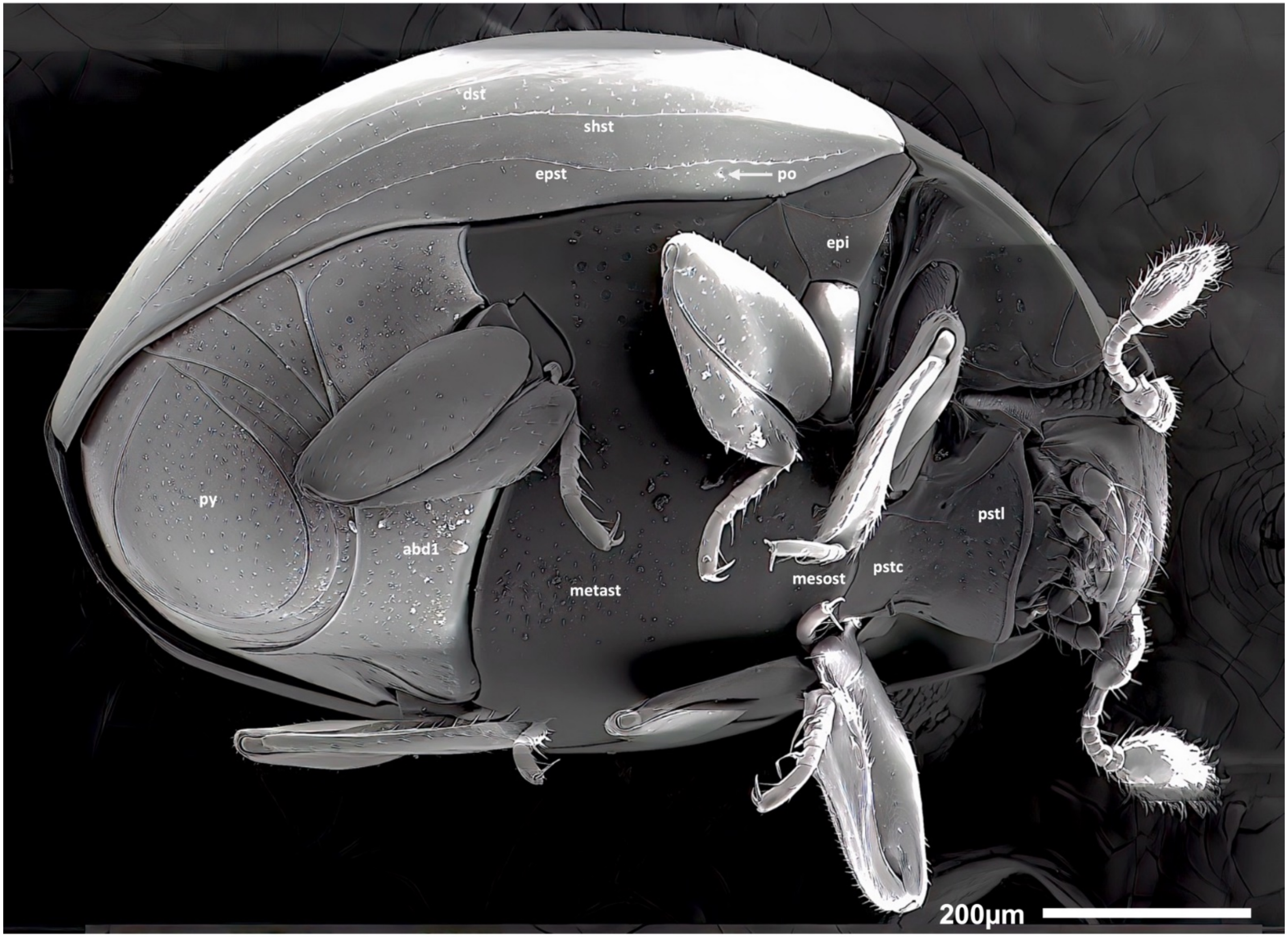

Figure S2. SEM micrograph of the ventro-lateral view of *Bacanius neoponerae* n. sp. abd1: abdominal ventrite 1; dst: elytral dorsal stria; epi: epimeron with one epimeral stria; epst: epipleural stria; mesost: mesosternum; metast: metasternum; po: pore; pstc: prosternal carina; pstl: prosternal lobe; py: pygidium; shst: elytral subhumeral stria. (Photos: M. Elías-Gutiérrez & G. Pérez-Lachaud).

Classification trees have been widely used by ecologists because of their high classification accuracy and their ability to characterize complex interactions among variables[55]. The Random Forest algorithm combines numerous classification trees to produce more accurate classifications. The by-products of the Random Forest calculations include measures of the importance of explanatory variables. In the tree only those variables that have a significant influence on the partition are represented. Classification trees work by repeatedly splitting the response data into two groups. The split is determined by the single predictor that best discriminates among the data. The binary splits continue to partition the data into smaller and smaller groups, or *nodes*, until the groups are no longer homogeneous. Each group is characterized by a typical value of the response variable, the number of observations in the group, and the values of the explanatory variables that define it.

We present here the classification tree based on the binary response "presence/ absence of histerids in *N. villosa* colonies". Figures in the squares are the proportion of the variable "histerid presence". The data are distributed according to the predictive values defined below the squares. For example, at the root of the tree, 86% of the colonies with histerids were found in sites with altitude below 261 m asl, and 14% at altitudes above this threshold. The probabilities of belonging to the different classes are estimated by the proportions that the model predicts in each class.

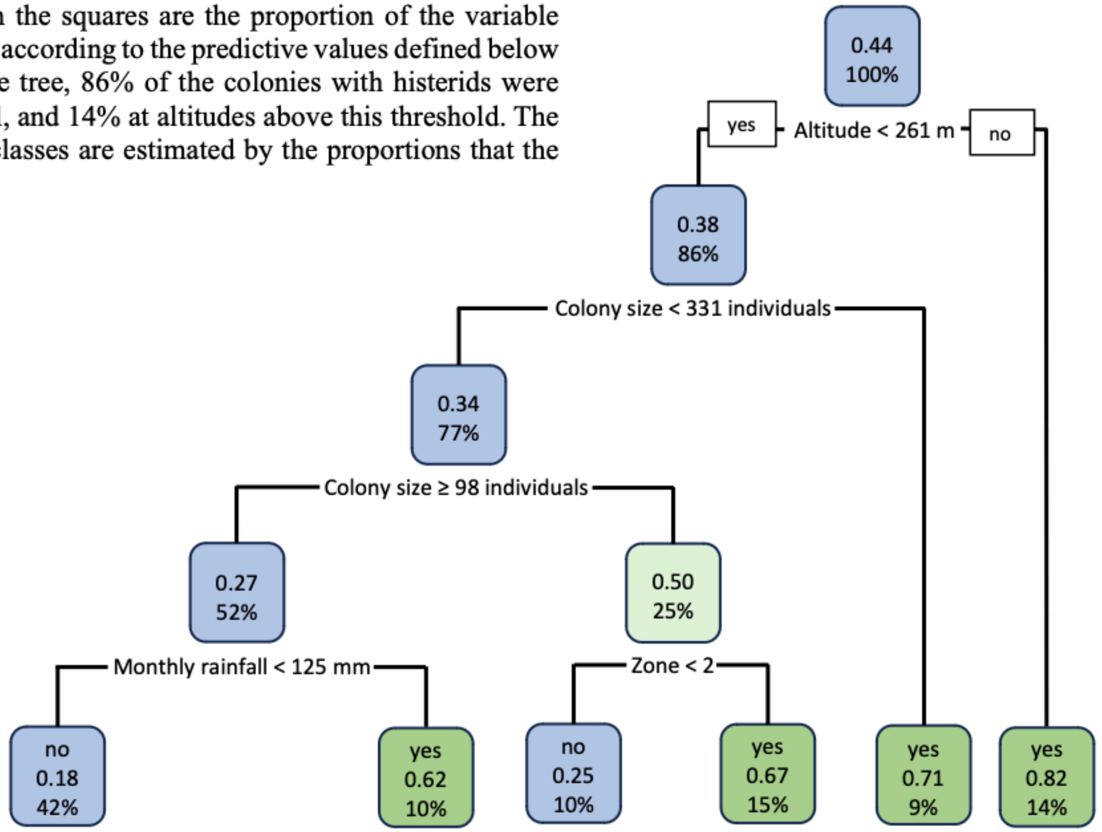

Figure S3. Drivers of *Bacanius* presence in *N. villosa* colonies. The classification tree shows how the data are distributed according to the most important variables predicting the association of *B. neoponerae* with *N. villosa* colonies (altitude of the sampling site, colony size, monthly rainfall and collecting zone). The response variable is binary (presence/absence) with 0.44 of ant colonies hosting the beetle (relative proportion 100%). Squares indicate the relative proportion of data belonging to each group (i.e., the number of observations in a group). The values of the explanatory variables used for the partition data are indicated below the squares. Zone 1: Sian Ka'an; Zone 2: Nuevo Becal; Zone 3: Ejido Blasillo.

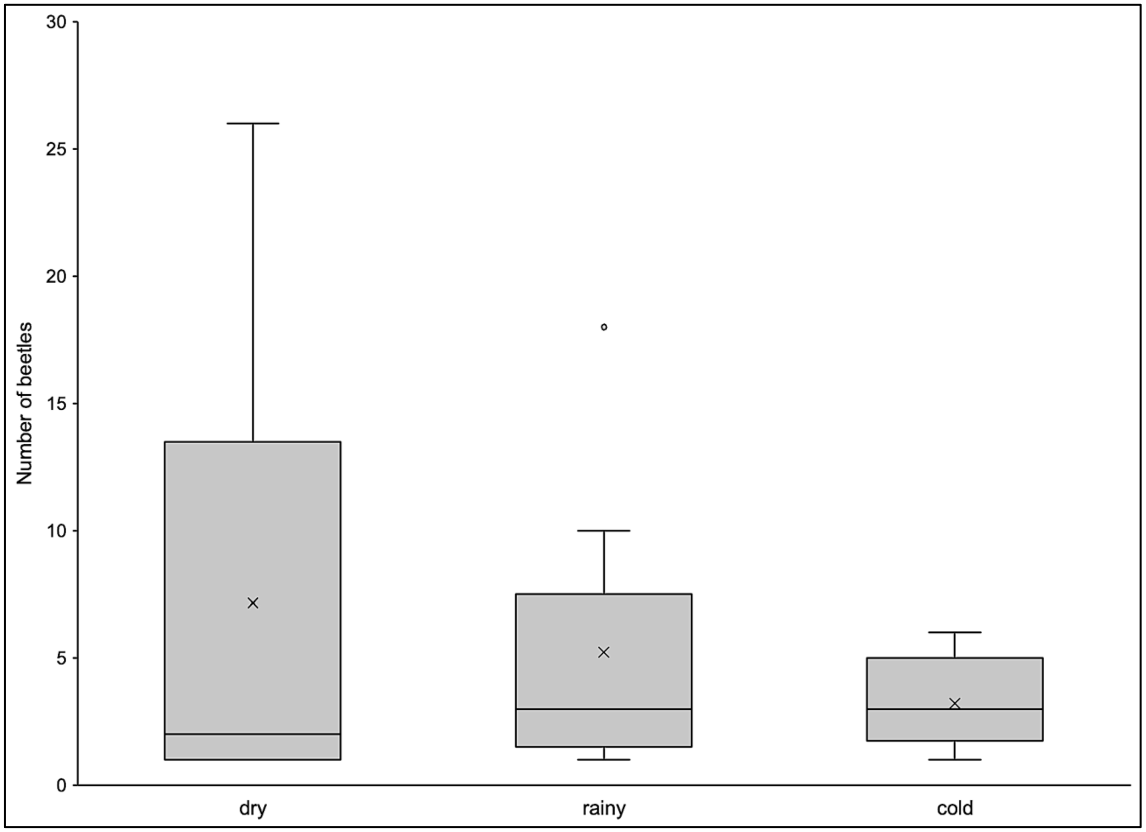

Figure S4. Boxplot of the number of *Bacanius neoponerae* found in *N. villosa* colonies nesting in *Aechmea bracteata* across different seasons (dry, rainy, cold) in southeastern Quintana Roo, Mexico (n = 12, 8, 14 nests, respectively). Each box shows upper and lower quartiles along with maximum and minimum (whiskers), median (thick line) and outliers.
